# Multifocal primary musculoskeletal tuberculosis in nonimmunocompromised patient from nonendemic area

**DOI:** 10.1259/bjrcr.20190077

**Published:** 2020-09-29

**Authors:** Hosameldeen Mostafa Ali, Hoda Mohamed Abdelaziz Shoshan

**Affiliations:** 1 Department of Radiology, Benha University, City, Egypt; 2 Department of Pathology, Benha University, City, Egypt

## Abstract

Iliopsoas primary tuberculous abscess is a rare clinical and imaging entity. Most reported imaging literature cases are secondary to tuberculous spondylodiscitis. Iliopsoas tuberculous inflammation and abscess constitutes a diagnostic challenge owing to its insidious onset and subtle non-specific symptoms. Here, in a case of right iliopsoas and thigh primary tuberculosis abscess complicated with right iliac bone osteomyelitis extended to the right hip joint. The conventional radiography, thigh and pelvic ultrasonography, MRI and CT examinations showed the whole right iliopsoas and thigh abscess compartments and right iliac bone osteomyelitis. No defined other pulmonary or abdominal tuberculous lesions. Percutaneous drainage of the thigh compartment under ultrasound guidance and microbiologic culture of the drained fluid elicited mycobacterium tuberculosis.

## Background

Extrapulmonary tuberculosis, describes mycobacterium tuberculosis (TB) invasion of organs systems other than the lung parenchyma. It has insidious onset and non-specific clinical findings. Clinical presentation varies according to the involved organ. Multiple organs could be involved at the same time.

Musculoskeletal TB represents about 1–3% of tuberculous involvements. Tuberculous spondylitis is the commonest form of the musculoskeletal TB (about 50%). Extraspinal musculoskeletal TB is less common. The average frequency of peripheral tuberculous arthritis is 60%.^1^ The average frequency of tuberculous osteomyelitis 38%,^2^ and of tuberculous tenosynovitis and bursitis is 2%.^3^

Haematogenous or lymphatic dissemination from a primary or reactivation infection focus carries the bacilli to involve the muscles, bones and joints.^4^

Striated muscles TB is a scarce form of musculoskeletal TB. Tuberculous myositis rarely encountered in immuncomptent individuals. Striated muscles are resistant to mycobacterial infection owing to its scanty oxygen concentration, lactic acid high concentration, and indigence of reticuloendothelial system elements.^5^

Pus and cellular debris collection within the fascia enclosing the iliopsoas complex constitutes the iliopsoas abscess.

Iliopsoas abscess has two distinctive variants. The primary infrequent variant results from a remote infection focus carried out via haematogenous dissemination and lymphatic spread. The secondary more frequent variant results from local spread of inflammatory or neoplastic process such of adjacent structures as vertebrae, kidneys, bowel loops and pancreas into the iliopsoas complex.

Primary iliopsoas compartment abscess in absence of preceding pulmonary TB or thoracolumbar tuberculous spondylodiscitis is a rare clinical and imaging entity.^6^

The current case describes an unusual multifocal primary musculoskeletal TB presented with right iliopsoas and thigh abscess, iliac bone osteomyelitis and hip early synovitis without an underlying disorder or preceding pulmonary TB.

## Case report

A 31-year-old female noticed a painless barely palpable right mid-front thigh subcutaneous swelling. There was no relevant medical history. No history of chronic debilitating disease. No family history of TB.

The clinical examination elicited subtle discoloration of the skin overlying the mid-thigh swelling. The patient was of average built. The vital signs were within normal range. There were no enlarged inguinal lymph nodes. The rest of the systemic examination was unremarkable.

**Figure 1.**
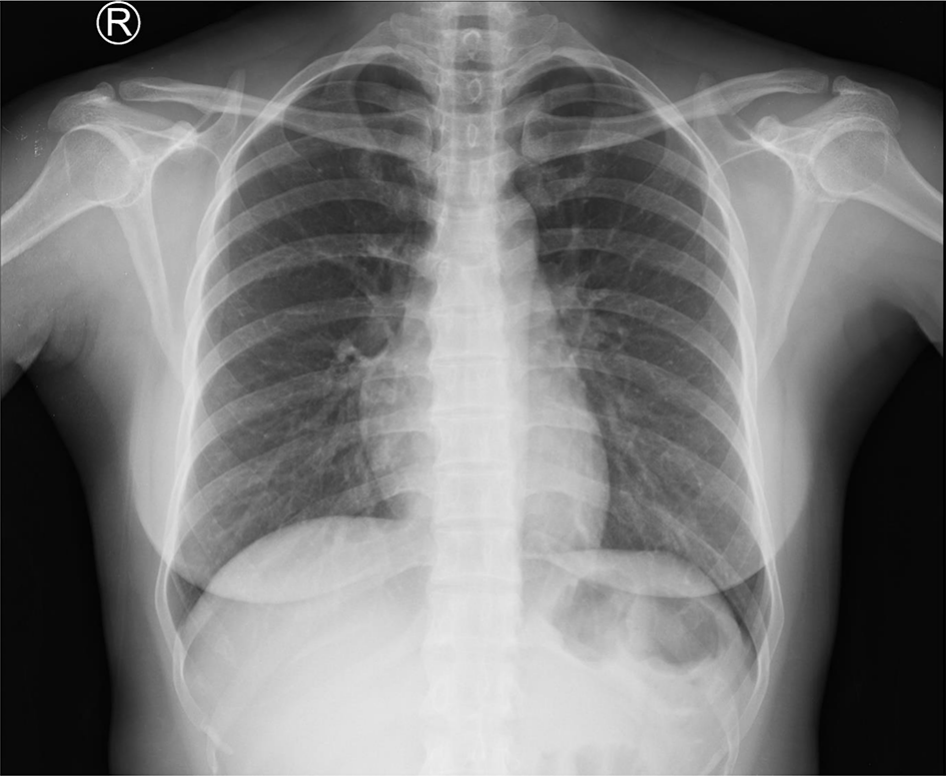
Conventional X-ray chest posteroanterior projection showed no evidence of thoracic tuberculosis.

**Figure 2.**
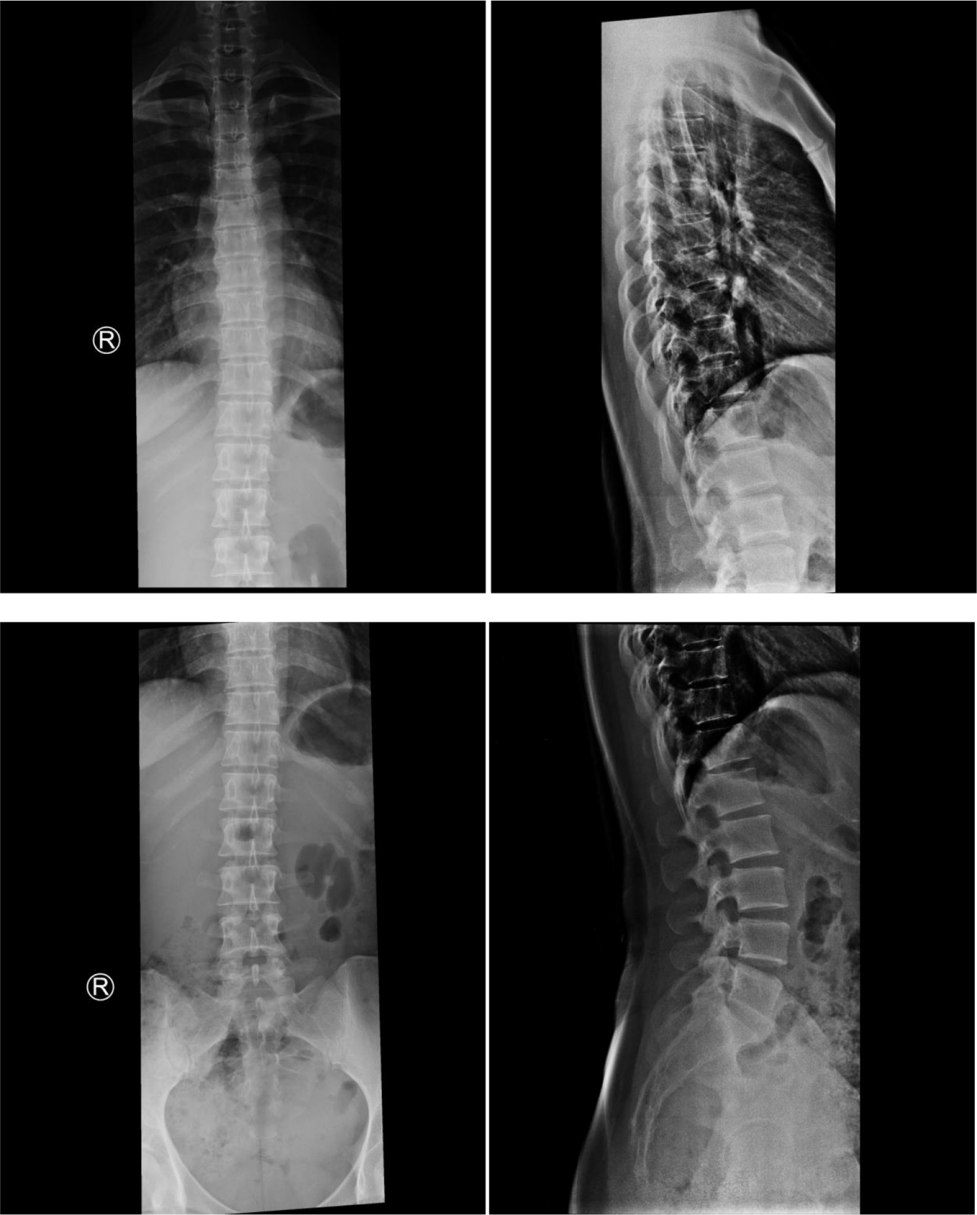
Conventional X-ray examinations of the thoracolumbar spine anteroposterior and lateral projections, showed no evidence of tuberculous spondylodiscitis.

Conventional X-ray examinations of the chest (Figure 1) and thoracolumbar spine (Figure 2) were normal.

**Figure 3.**
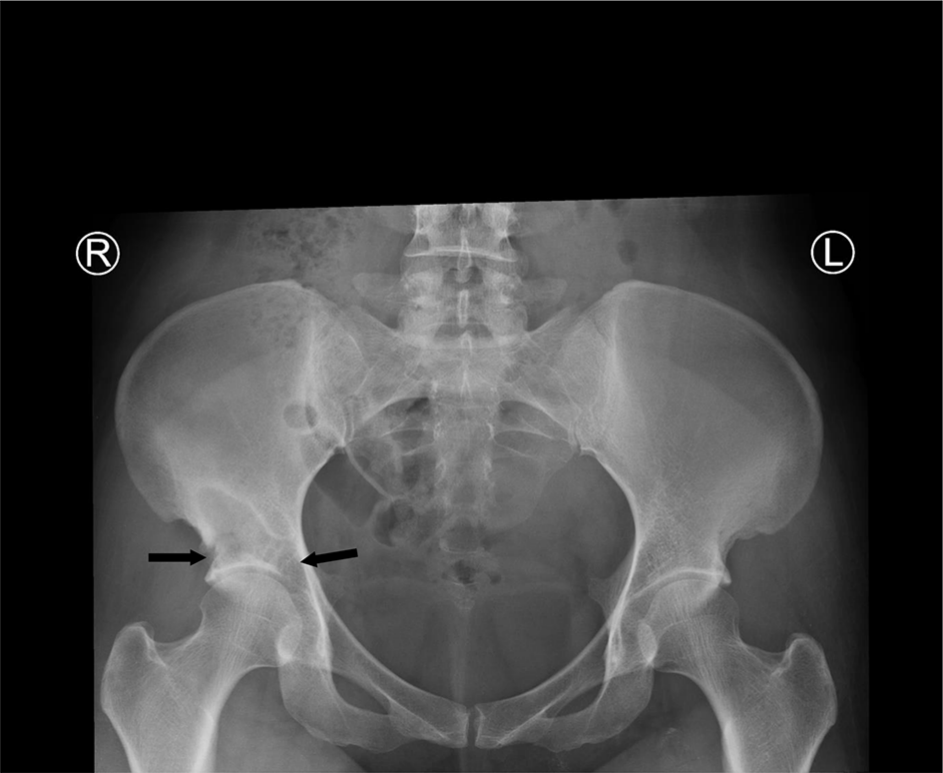
Conventional X-ray pelvis anteroposterior projection revealed right lower iliac bone irregular osteolytic areas with well-defined sclerotic rim. The osteolytic areas involved the superolateral and the superomedial aspects of the right acetabulum (black arrows) and likely breached to the acetabular articular surface.

Conventional X-ray examination of the pelvis (Figure 3) showed osteolytic areas involved the superolateral and the superomedial aspects of the right acetabulum and likely breached to the acetabular articular surface.

**Figure 4.**
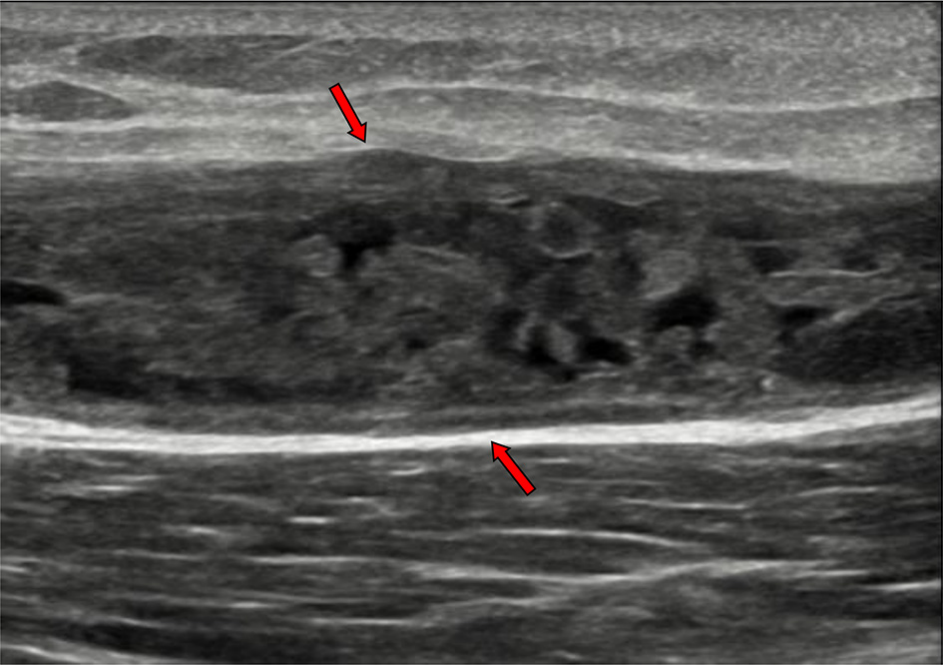
Right thigh anterior aspect longitudinal ultrasonography showed subcutaneous large elongated thick-wall cystic lesion (red arrows) contains turbid tenacious fluid.

**Figure 5.**
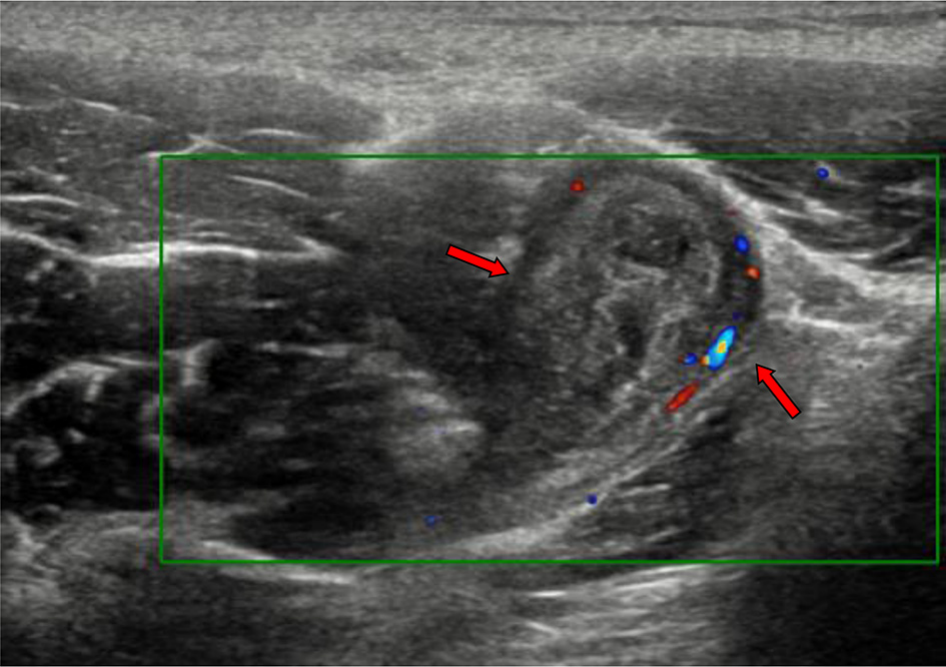
Longitudinal ultrasonography with colour Doppler of the upper right thigh showed another thick-wall cystic lesion involved the insertion of the iliopsoas complex (red arrows).

High-resolution ultrasonography of the right thigh (Figure 4) demonstrated a large oblong-shape subcutaneous and intermuscular cystic structure (about 195 × 60 × 24 mm) at the mid-thigh and extends upwards between the tensor fascia lata, rectus femoris and the vastus lateralis muscles. The right thigh cystic lesion seen continuous with another oblong-shape right iliopsoas (Figure 5) compartment cystic lesion (about 115 × 47 × 24 mm). Both right thigh and right iliopsoas cystic lesions have thick-walls and turbid tenacious fluid content.

Abdominal ultrasound examination showed no renal masses or perinephric collections. No defined intestinal masses or calcifications. Pelvic ultrasound examination showed no tubo-ovarian masses or abscesses.

**Figure 6.**
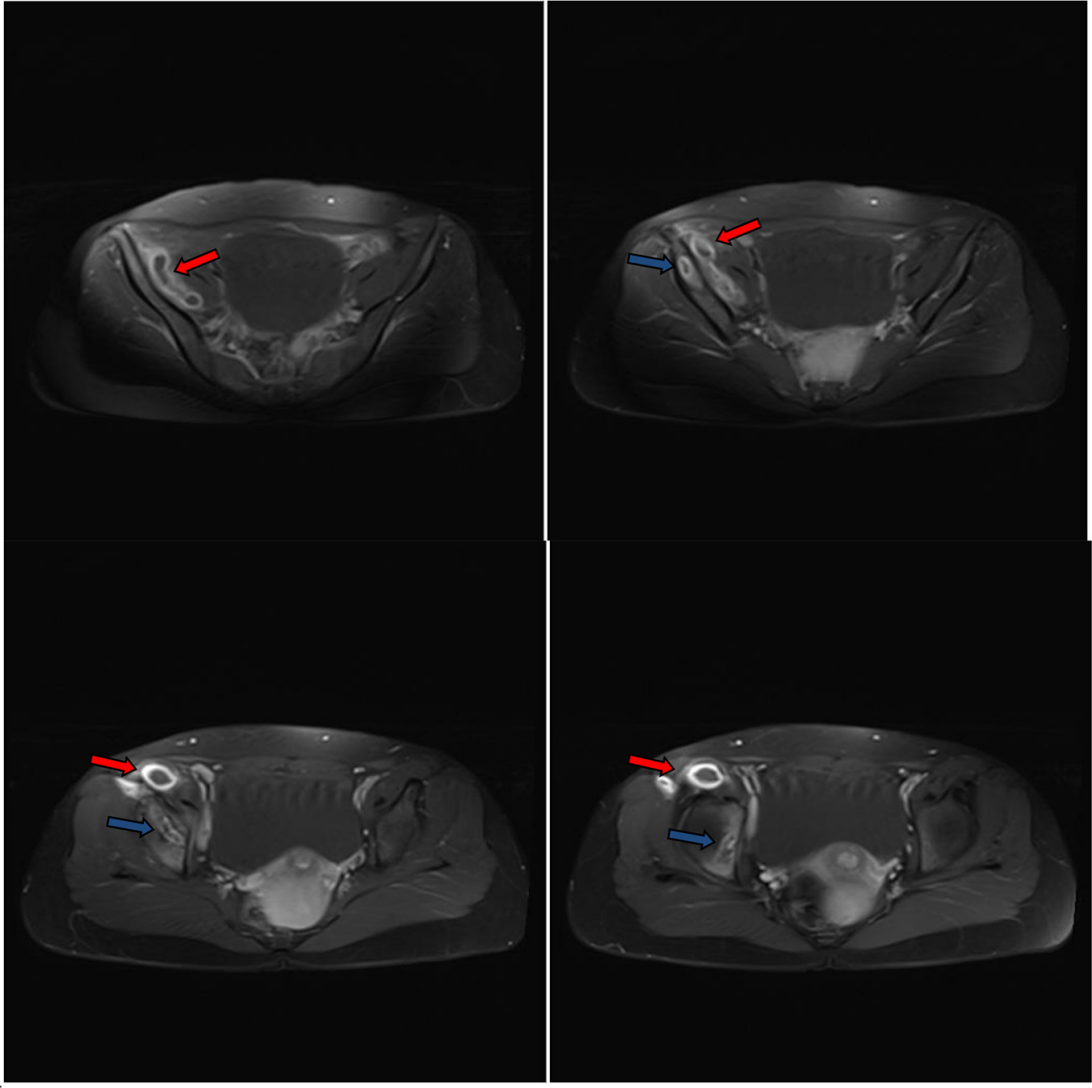
Post-contrast consecutive axial *T*_1_ images through the pelvis showed the right iliopsoas and right thigh abscess and lower right iliac bone osteomyelitis. Diffuse wall thickening and enhancement of the abscess walls (red arrows). Diffuse enhancement of the margins of the lower right iliac bone osteomyelitis (blue arrows). The abscess cavity contains hypointense fluid-equivalent signal. No intra-abscess septa, soft tissue mural nodules or gross calcifications.

**Figure 7.**
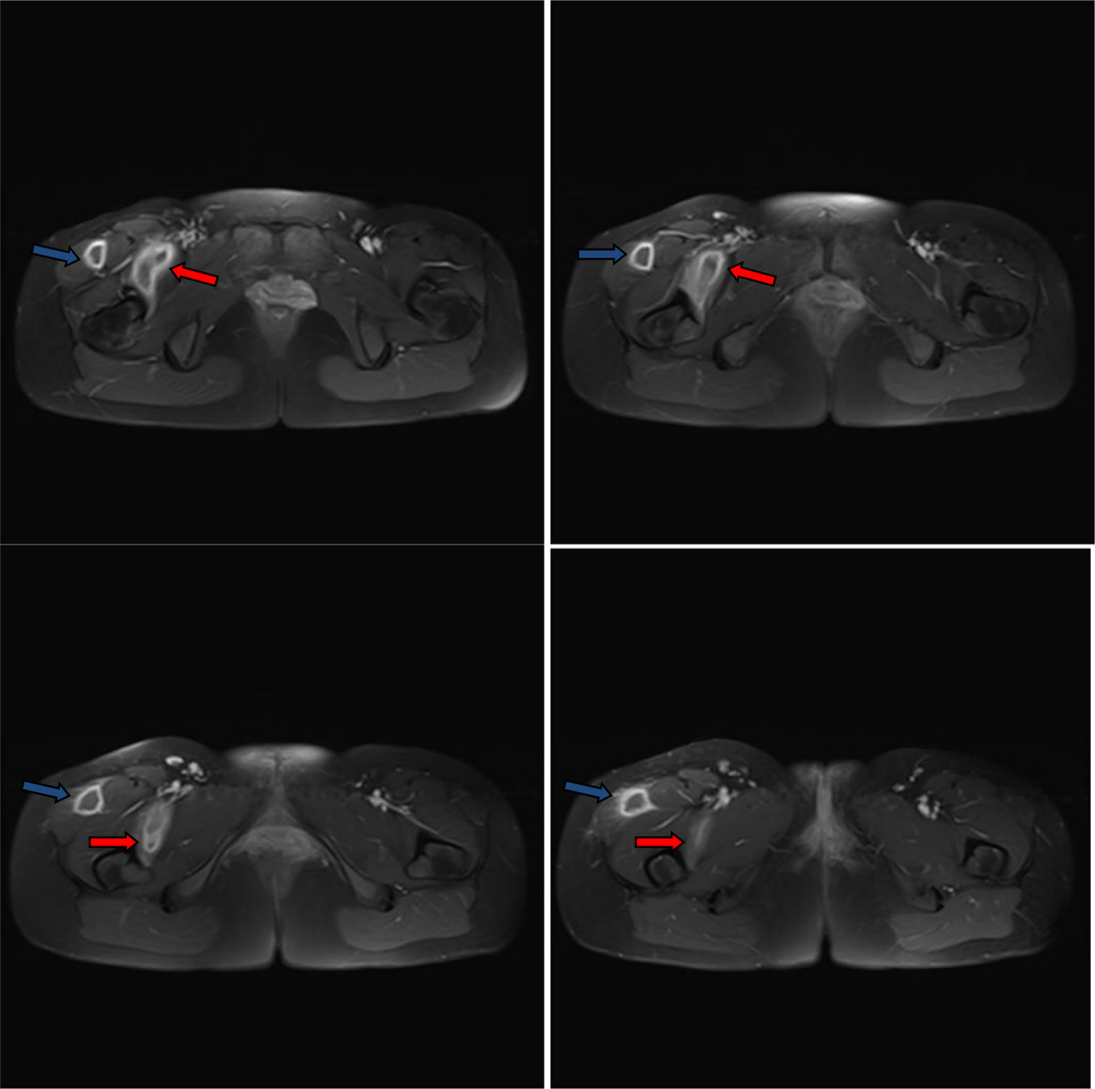
Post-contrast consecutive axial *T*_1_ images through the lower pelvis and proximal thigh showed the right iliopsoas (red arrows) and thigh abscess (blue arrows) compartments. Diffuse wall thickening and enhancement of the abscess walls. The abscess cavities contain hypointense fluid-equivalent signal. No soft tissue mural nodules or gross calcifications.

**Figure 8.**
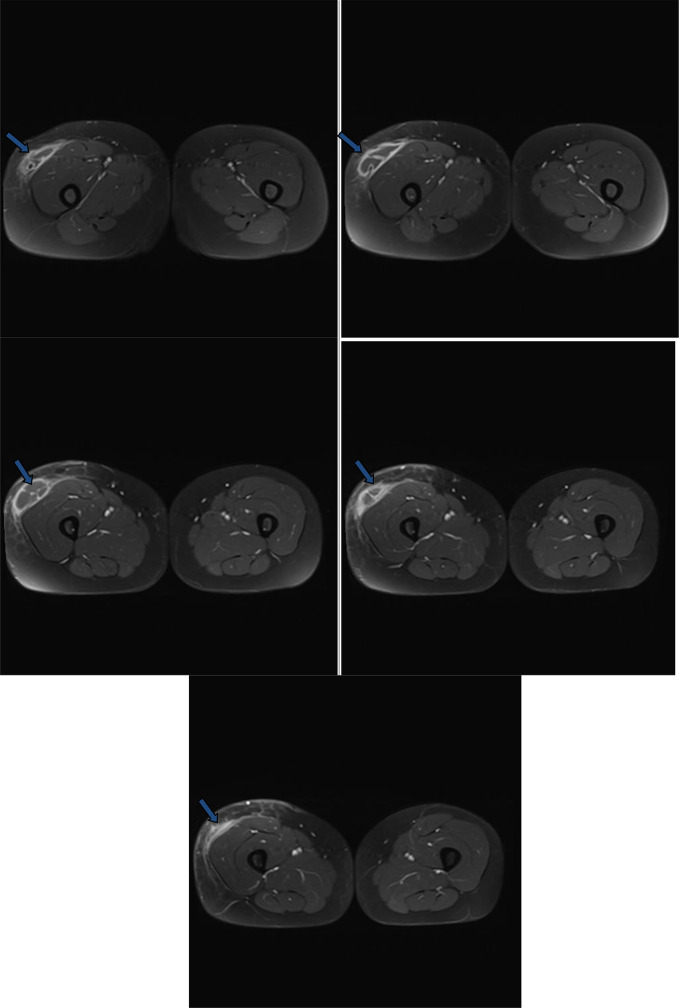
Post-contrast consecutive axial *T*_1_ images through the mid-right thigh showed the right thigh compartment of the abscess (blue arrows). The abscess is subcutaneous. Diffuse wall thickening and enhancement of the abscess walls. The abscess cavity contains hypointense fluid-equivalent signal. Few enhanced incomplete internal septa exist. No soft tissue mural nodules or gross calcifications.

**Figure 9.**
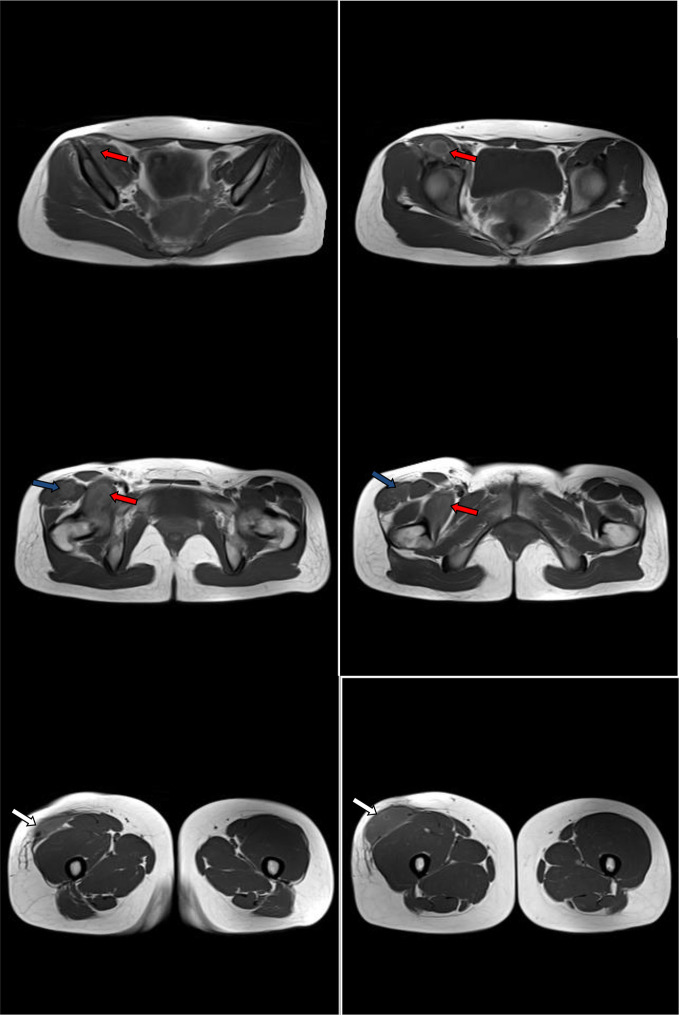
Non-contrast consecutive axial *T*_1_ images through the pelvis, proximal and mid-thigh showed the right iliopsoas and right thigh compartments of the abscess. The right thigh compartment is intermuscular at the proximal thigh (blue arrows) and subcutaneous at the mid-thigh (white arrows). The abscess cavities contain hypointense fluid-equivalent signal (red arrows). No soft tissue mural nodules or gross calcifications.

**Figure 10.**
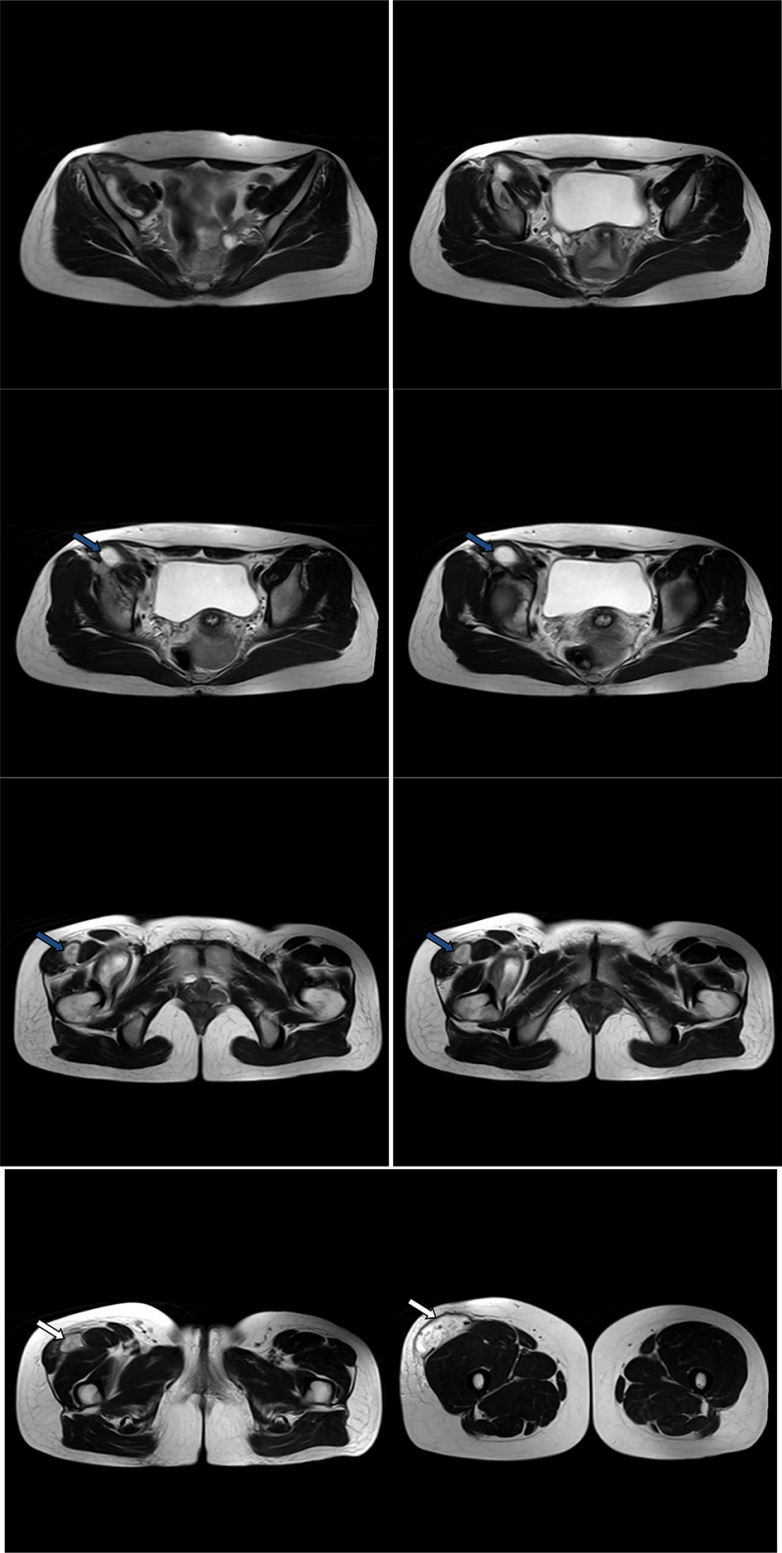
Non-contrast consecutive axial *T*_2_ images through the pelvis, proximal and mid-thigh showed the right iliopsoas and right thigh compartments of the abscess. The right thigh compartment is intermuscular at the proximal thigh (blue arrows) and subcutaneous at the mid-thigh (white arrows). The abscess cavities contain hyperintense fluid-equivalent signal. No soft tissue mural nodules or gross calcifications. Distal thigh compartment showed incomplete internal septa.

**Figure 11.**
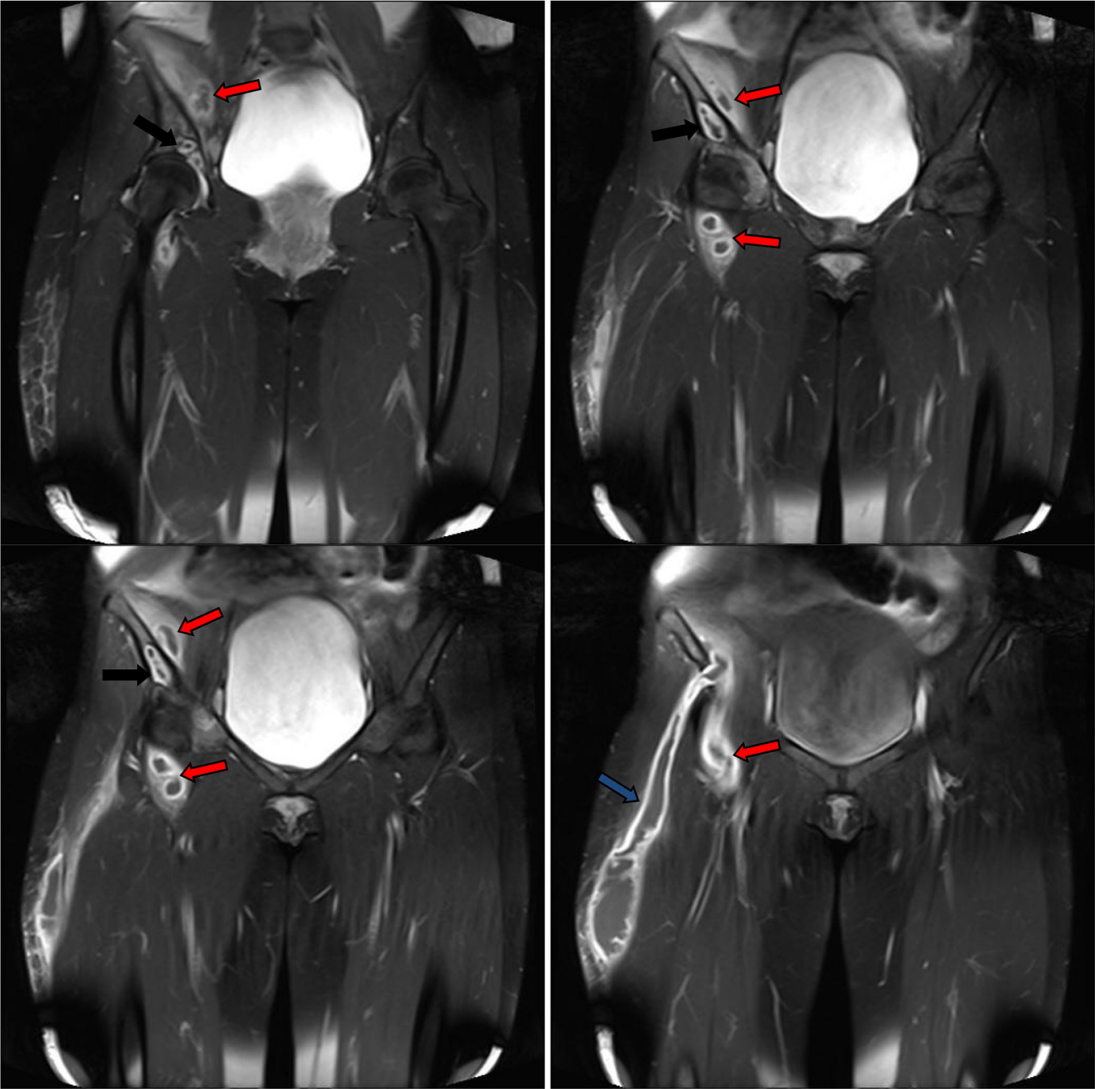
Post-contrat consequtive coronal *T*_1_ images showed thickened enhanced walls of the iliopsoas (red arrows) and right thigh (blue arrow) abscess compartments. No soft tissue mural nodules. Incomplete septa at the distal thigh compartment. Enhanced margins of the right iliac bone osteomylitis (black arrows).

**Figure 12.**
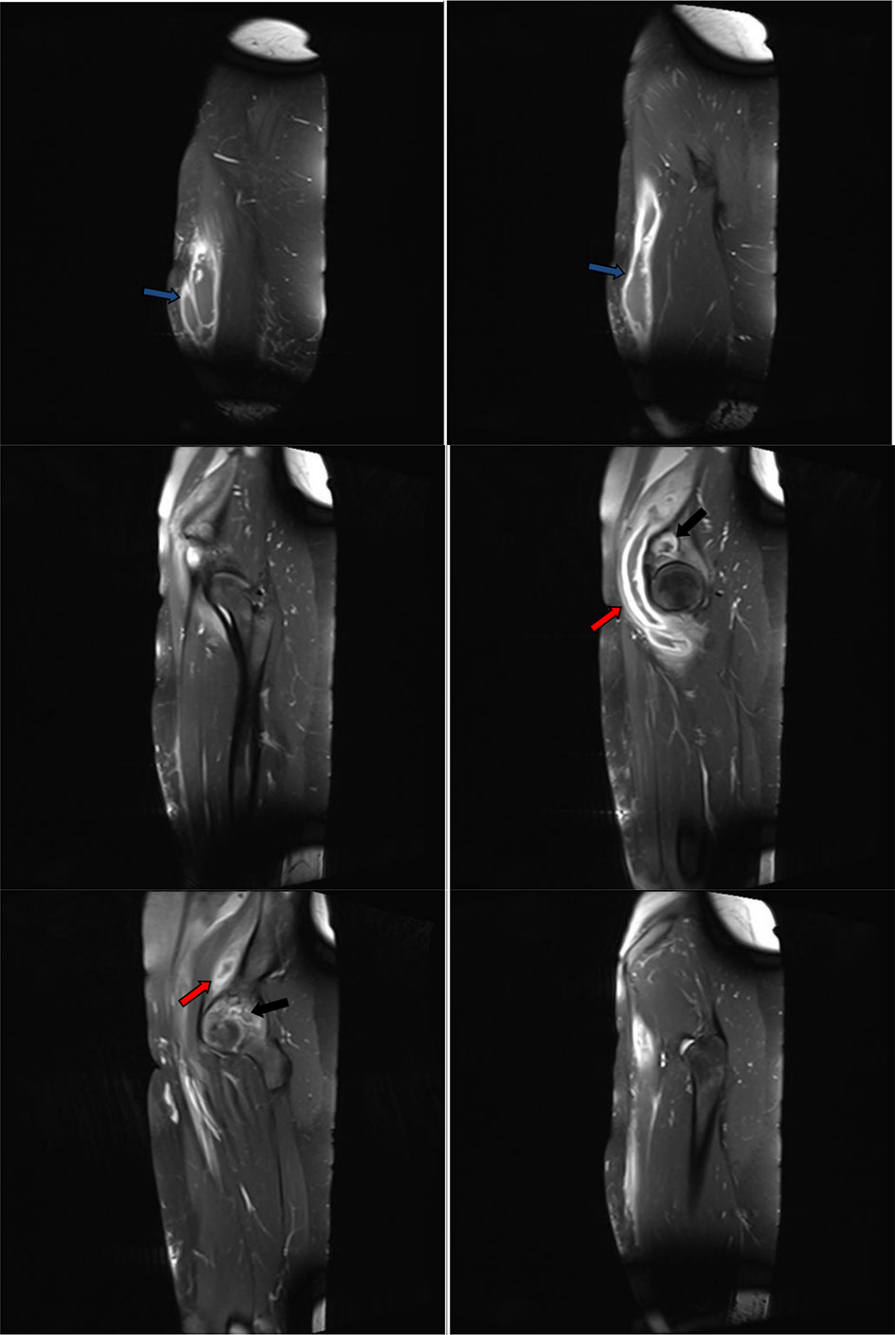
Post-contrat consequtive sagittal *T*_1_ images showed thickened enhanced walls of the iliopsoas (red arrows) and right thigh (blue arrows) abscess compartments. No soft tissue mural nodules. Incomplete septa at the mid-thigh compartment. Enhanced margins of the right iliac bone osteomylitis (black arrows).

Pre- and post-i.v.-contrast MR examination of the pelvis and right thigh examination performed and the images (Figures 6–12) elicited a large oblong-shape enhanced walls cystic lesion extending from the right iliac fossa to the lesser trochanter down to below the mid-right thigh. Moreover, the MR images showed distal right iliac bone and acetabulum roof cystic areas (about 26 × 26 × 15 mm) with enhanced margins. Those cystic areas likely penetrated the acetabular roof to the right hip synovial cavity.

**Figure 13.**
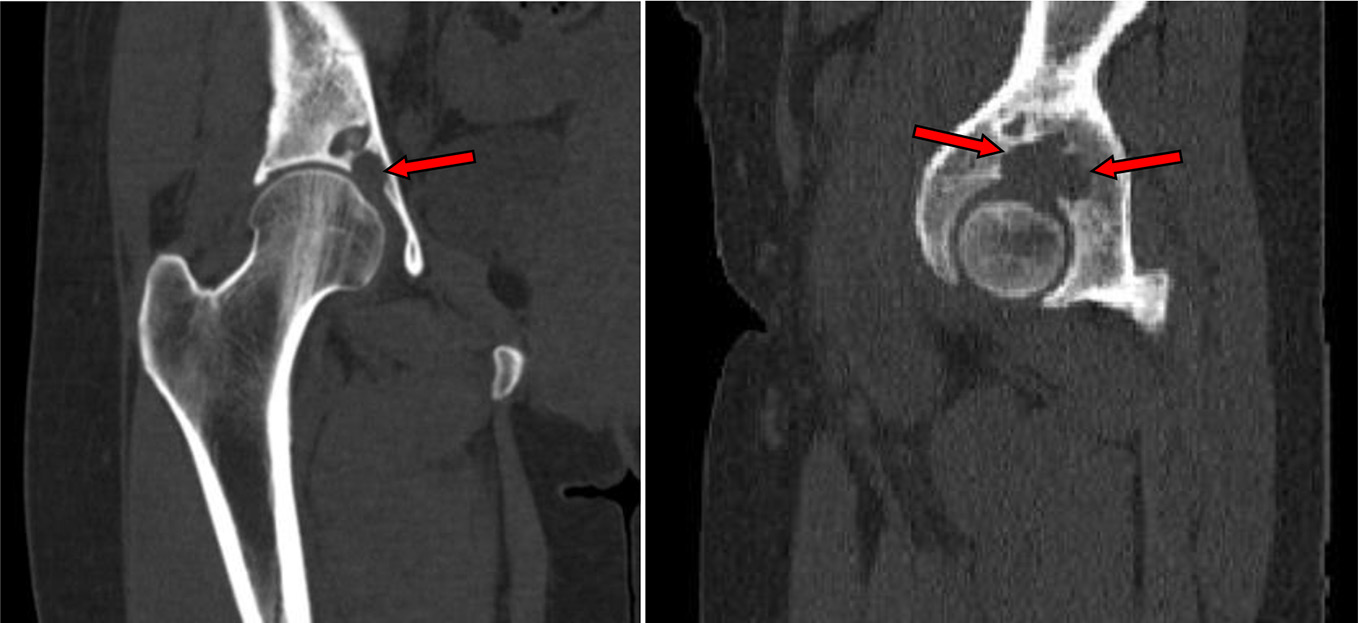
Non-contrast CT coronal and sagittal reformatted images showed the lower right iliac bone osteolytic lesions (red arrows) eroded the acetabular articular surface to extend to the right hip joint.

**Figure 14.**
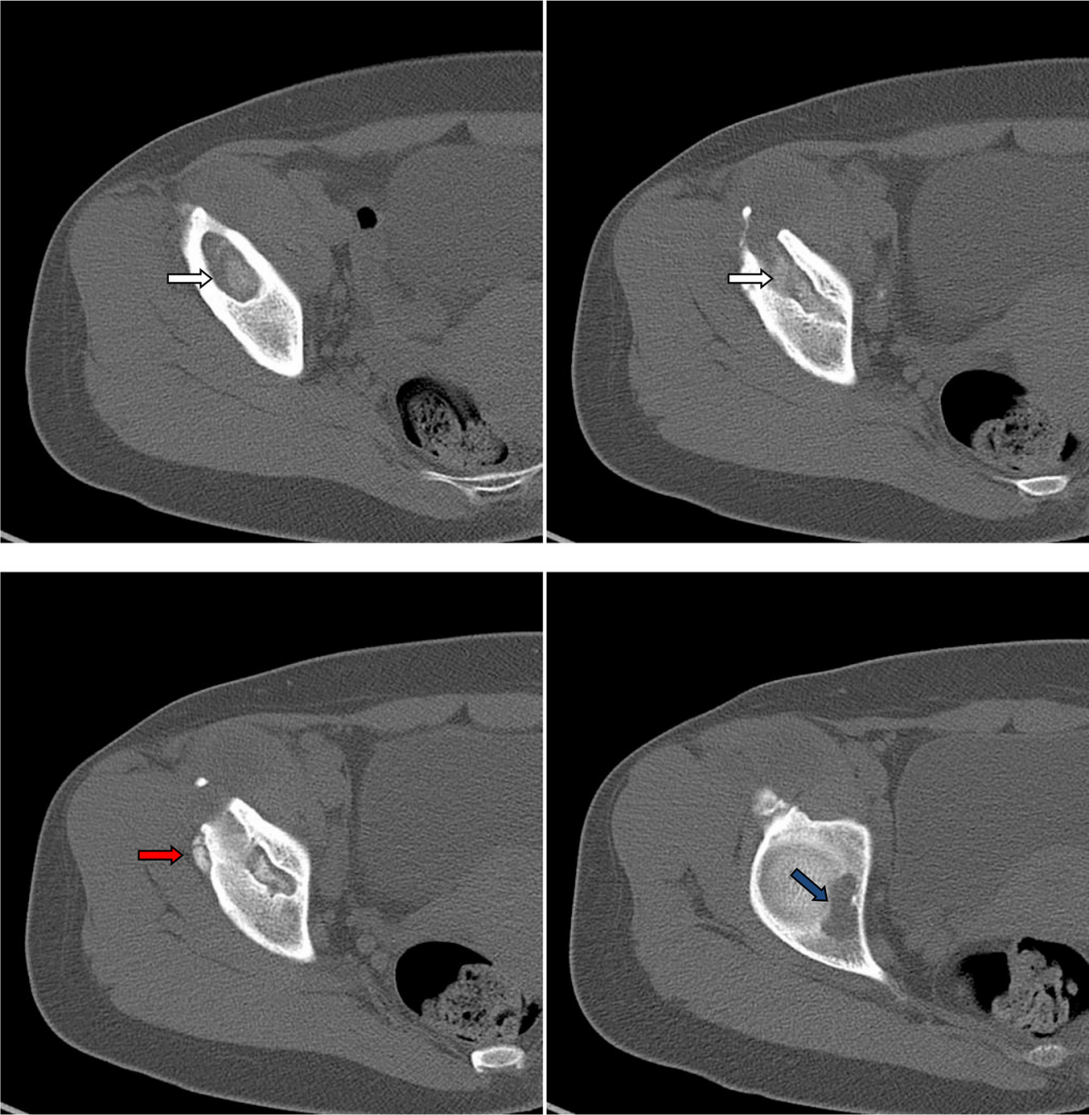
Non-contrast consecutive axial CT bone window images through the right lower iliac bone and hip joint showed bone fragments (red arrow) and osteolytic lesions (white arrows) eroded the bone down to the acetabular articular surface and hip joint cavity (blue arrow).

Non-contrast CT (Figures 13 and 14) performed to clarify the bone involvement. The CT images showed cystic lesions at the lower right iliac bone with defect and fragmentation at the anterior aspect of the bone underneath the right iliacus muscle. No defined gross calcifications. No regional inguinal or iliac lymphadenopathy.

The haematologic laboratory work-up showed an elevated leukocytic count (mainly neutrophilia), C-reactive protein and erythrocyte sedimentation rate. Human immunodeficiency virus and viral hepatitis antigens/antibodies/DNA were not detectable in serum. Other blood investigations were within the reference range. The acid-fast bacilli smear of two sputum samples was negative under fluorescent microscopy.

Diagnostic aspiration from the right thigh abscess was performed under ultrasound guidance revealed a straw colored fluid. The bacterial culture and Gram staining were negative. Blood cultures were sterile. The fluid acid-fast staining came positive, and the culture developed mycobacterium TB.

The patient underwent ultrasound-guided percutaneous drainage of the thigh compartment and began combination antituberculous therapy.

## Discussion

The mycobacterium TB is basically an airborne droplet infection is that gets into lungs. The vast majority of the bacilli are engulfed by the alveolar macrophages and demolished. The macrophages engulfed the mycobacterium then surrounded by T-cell lymphocytes creating granulomas. The granulomas have paucity of nutrients that subside mycobacterial growth and therefore hinder the infection. The patients with disabled immune system cannot control the infection and develop primary pulmonary infection. Mycobacterial spread to other organ systems occurs when the bacilli get its way to the blood of lymphatic vessels.

The increased number of patients with immunity-depressive diseases, wider use of immunosuppressive drugs, aging population and drug-resistant mycobacterium TB strains resulted in increased TB incidence.

Any organ or body system could be the seat of TB infection. The majority of extrapulmonary TB cases involve the pleura, musculoskeletal and lymphatic systems. Most cases proved to have previous pulmonary tuberculous focus.^7^

TB bacilli from a pulmonary infection focus invade the pleural cavity and then migrates through the lymphatics and blood vessels to non-thoracic organs developing extrapulmonary TB.

Tuberculous osteomyelitis represents about 1–2% of all cases of TB and about 10% of extrapulmonary TB.^8^

Multifocal musculoskeletal TB rarely encountered in nonimmunocompromised individuals from nonendemic areas with no prior pulmonary involvement.^9^

The lumbar and thoracic spine are the most common sites for musculoskeletal TB and account for more than about 40–60% cases. The crvical spine involvement is uncommon and accounts only for 2–3% of cases.^10^

Extrapulmonary TB accounts for about 15–20% of all TB cases. It should be anticipated in cases presented with pleural effusion and pleural wall thickening, chronic lymphadenopathy, monoarthritis with negative bacterial cultures, and thoracic spine spondylodiskitis and concomitant HIV infection.^11^

The iliopsoas compartment encompasses the psoas major, psoas minor (if exists) and iliacus muscles. The psoas major muscle origins from the transverse processes of the distal thoracic and all lumbar vertebrae. The iliacus muscle arises from the iliac fossa. The psoas major and iliacus muscles join and the common tendon inserts into the femoral lesser trochanter. The transversalis fascia lies anterior to the psoas major. The transversalis fascia constitutes the posterior boundary of the retroperitoneum.

The iliopsoas compartment is enveloped within a strong fascia which has numerous connections with the adjacent structures. Superiorly, iliopsoas fascia blends with the thoracic fascia as the psoas major passes underneath the diaphragmatic arcuate ligament. Infection can spread from the chest to the abdomen and vice versa through the psoas major muscle origin. The inferior aspect of the psoas fascia joins fascia lata of the thigh.

The iliopsoas fascia represents the posterior boundary of the posterior pararenal space. It merges inferiorly with the posterior renal fascia. The anterior and posterior renal fasciae does not always fuse inferiorly. This is a potential route of disease spread to and from the posterior pararenal space and kidneys to the psoas compartment. The iliospoas compartment is related posteriorly to the quadratus lumborum muscle and thoracic and lumbar spine.^12^

The iliopsoas compartment is profusely vascularized and lies close to the retroperitoneal lymphatics which contributes to its susceptibility to haematogenous and lymphatic seedling and infection. Its vicinity to the retroperitoneal structures, vertebrae, iliac bones and hip joints explains feasibility of infection spread to and from these structures.

As a retroperitoneal muscle that originates from the lateral borders of the 12th thoracic to fifth lumbar vertebrae, and ends as a tendon that inserts into the lesser trochanter the psoas muscle is a retroperitoneal muscle that originates from the lateral borders of the 12th thoracic to fifth lumbar vertebrae, and ends as a tendon that inserts into the lesser trochanter.

Secondary iliopsoas infection or abscess predominantly likely results from direct extension from a nearby organ. The less frequent primary iliopsoas abscess results from either haematogenous or lymphatic spread usually from a cryptic septic or tuberculous focus. Spondylodiskitis, diverticulitis, pyelonephritis and perirenal abscess represent the probable local causes of the secondary variant. Commonest causative organisms are *Staphlococcus aureus* (about 88%), *Streptococci* (about 5%) and Gram negative bacilli (about 3%).^13^

Iliopsoas compartment primary tuberculous abscess with occult cause rarely encountered in the clinical practice and is usually idiopathic.

The presence of more immune-compromised patients secondary to HIV infection, cancer, cancer treatment, organ-transplant patients receiving immunosuppressive agents and longstanding diabetes increases the incidence of musculoskeletal TB.^14^

Iliopsoas compartment TB has indolent nature usually stays clinically silent for long time. It clinically presents with a variety of non-specific symptoms as fever, chills, easy fatigability, weight loss, loin pain and hip movement restriction.

Tuberculous osteomyelitis presents a granulomatous lesion with caseation and bone necrosis. Joint implication through transphyseal spread of inferior. Multifocal synchronous musculoskeletal (bones, muscles, tendons and joints) involvement may be seen, due to haematogenous spread, with seeded bacilli at different times and sites. Suppressed host immune response predisposes to multifocal disease pattern.^15^

The probable conventional radiography findings of iliopsoas compartment TB include obscured or distorted iliopsoas margin, iliopasoas gas lucency, soft tissue calcifications, vertebral bone erosions and hip bone and scoliosis. The current case showed distal right iliac bone osteolytic areas with well-defined sclerotic rim eroded the superior acetabulum to the right hip joint. No gross calcifications of the iliopsoas compartment or the thigh.

Chest conventional radiography could elicit evidence of prior TB infection. The negative chest radiography does not exclude extrapulmonary musculoskeletal TB.

On conventional radiography, tuberculous osteomyelitis presents as osteolytic areas, bone erosions and periarticular osteoporosis. Sclerosis is seldom depicted. In comparison to pyogenic osteomyelitis, sequestration is infrequent and less extensive in tuberculous osteomyelitis. CT is superior to conventional radiography for assessment of sequestrum formation.^16^ The elicited bone lesions are well-defined and hypodense with no surrounding reactive sclerosis.^17^

Ultrasonography has no appreciable value in evaluating tuberculous osteomyelitis unless there is associated tuberculous myositis or arthritis.

MRI is preferable to other imaging modalities concerning tuberculous osteomyelitis. Marrow changes are demonstrated as areas of low and high signal intensity on *T*_1_W and *T*_2_W weighted images, respectively. The lesions exhibit marginal enhancement post-gadolinium chelates i.v. administration. Areas of necrosis appear hyperintense on *T*_2_ weighted images and lacking enhancement on *T*_1_ weighted images.

Ultrasonography is the primary investigation of tuberculous myositis and bursitis. It reveals the degree and extent of muscles and tendons involvement.

Retroperitoneal space ultrasonographic examination can be hindered by bowel gases. Ultrasonography could be employed along with CT and MRI for evaluation of the possible extensions of the iliopsoas compartment abscess to the thigh. Ultrasonography can guide aspiration and drainage of the abscess or intracavitary injection of antibiotics.

Tuberculous myositis and tenosynovitis elicited as low *T*_1_ weighted signal and high *T*_1_ weighted MR signal. The iliopsoas abscess walls appears as linear *T*_1_ hyperintense and *T*_2_ hyperintense signal. Such configuration could be to oxygen free radicals and iron within macrophages in abscess wall of the abscess. Abscess wall peripheral rim enhancement seen after gadolinium contrast administration.^5^

CT outlines the location and possible extensions of the psoas compartment abscess. CT can identify the iliopsoas abscess predisposing pathology as perinephric abscess or bowel inflammatory lesions. CT could depict associated involvement of the vertebrae, pelvic bones or the femur. The iliopsoas abscess appears as areas of hypodensity and enlargement of the iliopsoas muscle. Ancillary CT findings include iliopsoas fluid densities, soft tissue gas densities obscured peripsoas fat planes and possibly adjacent bone erosions.

Non-contrast CT also could depict calcifications in chronic and treated cases. Contrast-enhanced CT optimizes visualization of the abscess extensions and margins.^18^

MRI could preferably differentiates iliopasoas compartment abscess from iliopasoas haematoma, primary or secondary tumours.^19^

Iliopsoas compartment primary (liposarcoma, fibrosarcoma, leiomyosarcoma and haemangiopericytoma) and secondary tumors are rare. The imaging features of these lesions have wide diversity overlappingapart from liposarcomas because of the presence of fat component depicted by MRI. The iliopsoas compartment abscess has predominant fluid intensity which could be readily differentiated from the solid tumours on *T*_1_ and *T*_2_ weighted MR images.

Iliopsoas compartment haematoma is either idiopathic or secondary. The proposed causes of iliopsoas secondary haematoma include bleeding trauma, bleeding diathesis or anticoagulant therapy. CT elicits acute iliopsoas haematoma as enlarged muscle compared to its contralateral one and fluid density. CT cannot differentiate iliopsoas abscess form chronic haematoma and necrotic tumours.

The MR appearance of iliopsoas haemorrhage relies primarily on the age of the haematoma and the used MR sequence (*T*_1_- or *T*_2_ weighted). As a haematoma ages, the haemoglobin passes through several forms: oxyhaemoglobin in the hyperacute phase, deoxyhaemoglobin in the acute phase, intracellular methaemoglobin in the early subacute phase, extracellular methaemoglobin in the late subacute phase, and haemosiderin in the chronic phase. Acute haematoma showed *T*_1_ signal iso- to hypointense to the muscle and *T*_2_ signal hypointense to the muscle. Subacute haematoma shows *T*_1_ hyperintense signal and *T*_2_ hypointense signal. As the haematoma ages the *T*_2_ turns hyperintense. Chronic haematoma showed *T*_1_ and *T*_2_ hypointense signals.^20^

The multimodality imaging should be employed to rule out other foci of infection or inflammation in the lungs, spine, pelvic bones, genitourinary or gastrointestinal tracts.

The multimodality imaging findings alone seldom allow to define the precise aetiology of iliopsoas compartment disease. The imaging findings should be combined with relevant history and clinical data to improve its diagnostic accuracy. Imaging is reliable for guiding iliopsoas abscess biopsy biopsies and drainage.

## Conclusions

Extrapulmonary TB can affect any organ through haematogenous and lymphatic routes. The depiction and characterization of extrapulmonary musculoskeletal TB probably is intractable considering its vague clinical presentation and indolent progression pattern. Primary musculoskeletal TB in absence of thoracic tuberculous lesions is further more convoluted. Combining imaging procedures be employed in order to narrow the differential diagnosis. The definitive diagnosis requires the isolation of TB bacilli through biopsies and cultures of the infected tissues.

Chest conventional radiography may elicit evidence of previous pulmonary or pleural infection. MRI is more sensitive than CT in evaluation of soft musculoskeletal TB, with more accurate delineation of inflammatory changes of the muscles, bones and synovial cavities. CT is accurate concerning bone involvement.

## Learning objectives

- Review the iliopsoas and retroperitoneal anatomy including, muscles, fascial planes, adjacent organs and routes of spread of infection.
- Outline the clinical presentation and probable source of iliopsoas compartment tuberculous abscess and right iliac bone osteomyelitis.
- To describe the conventional radiography, ultrasonography, CT and MRI criteria of rarely encountered iliopsoas compartment and right thigh primary tuberculous abscess and associated right iliac bone tuberculous osteomyelitis.
- To differentiate the iliopsoas compartment tuberculous abscess from other iliopsoas compartment pathologies as rare primary or secondary tumours or haematoma.

## References

[1] Suh JS, Lee JD, Cho JH, Kim MJ, Han DY, Cho NH Mr imaging of tuberculous arthritis: clinical and experimental studies. J Magn Reson Imaging 1996; 6: 185–9. doi: 10.1002/jmri.1880060133 8851426

[2] Martini M, Adjrad A, Boudjemaa A Tuberculous osteomyelitis. A review of 125 cases. Int Orthop 1986; 10: 201–7. doi: 10.1007/bf00266209 3771030

[3] Jaovisidha S, Chen C, Ryu KN, Siriwongpairat P, Pekanan P, Sartoris DJ, et al Tuberculous Tenosynovitis and bursitis: imaging findings in 21 cases. Radiology 1996; 201: 507–13. doi: 10.1148/radiology.201.2.8888250 8888250

[4] Tuli SM General principles of osteoarticular tuberculosis. Clin Orthop Relat Res 2002; 398: 11–19. doi: 10.1097/00003086-200205000-00003 11964626

[5] Batra S, Ab Naell M, Barwick C, Kanvinde R Tuberculous pyomyositis of the thigh masquerading as malignancy with concomitant tuberculous flexor tenosynovitis and dactylitis of the hand. Singapore Med J 2007; 48: 1042–6. 17975695

[6] Turunç T, Turunç T, Demiroğlu YZ, Colakoğlu S Retrospective evaluation of 15 cases with psoas abscesses. Mikrobiyol Bul 2009; 43: 121–5. 19334388

[7] Lin JN, Lai CH, Chen YH, Lee SS, Tsai SS, Huang CK, et al Risk factors for extra-pulmonary tuberculosis compared to pulmonary tuberculosis. The International Journal of Tuberculosis and Lung Disease 2009; 5: 1350–7. 19383196

[8] Rieder HL, Snider DE, Cauthen GM Extrapulmonary tuberculosis in the United States. Am Rev Respir Dis 1990; 141(2 I): 347–51. doi: 10.1164/ajrccm/141.2.347 2301852

[9] Habte-Gabr E, Jayabalan V, Tummala MR Usefulness of imaging in disseminated tuberculosis. Clin Nucl Med 2006; 31(no. 5): 303–6. doi: 10.1097/01.rlu.0000210546.35027.ab 16622346

[10] Özol D, Köktener A, Uyar ME Active pulmonary tuberculosis with vertebra and rib involvement: case report. South Med J 2006; 99(no. 2): 171–3. doi: 10.1097/01.smj.0000198642.28149.94 16509557

[11] Blumberg HM, Burman WJ, Chaisson RE, Daley CL, Etkind SC, Friedman LN, Etkind, Friedman S, et al American thoracic Society/Centers for disease control and Prevention/Infectious diseases Society of America: treatment of tuberculosis. Am J Respir Crit Care Med 2003; 167: 603–62. doi: 10.1164/rccm.167.4.603 12588714

[12] Taiwo B Psoas abscess: a primer for the internist. South Med J 2001; 94: 2–5. 11213936

[13] Muttarak M, Peh WC Ct of unusual iliopsoas compartment lesions. Radiographics 2000; 20 Spec No(suppl_1): S53–66. doi: 10.1148/radiographics.20.suppl_1.g00oc07s53 11046162

[14] Adelekan MO, Taiwo SS, Onile BA A review of psoas abscess. Af J Clin Exp Micro 2004; 5: 55–63. doi: 10.4314/ajcem.v5i1.7360

[15] De Vuyst D, Vanhoenacker F, Gielen J, Bernaerts A, De Schepper AM Imaging features of musculoskeletal tuberculosis. Eur Radiol 2003; 13: 1809–19. doi: 10.1007/s00330-002-1609-6 12942283

[16] Teo HEL, Peh WCG Skeletal tuberculosis in children. Pediatr Radiol 2004; 34: 853–60. doi: 10.1007/s00247-004-1223-7 15278319

[17] Morris B, Varma R, Garg A, Awasthi M, Maheshwari M Multifocal musculoskeletal tuberculosis in children: appearances on computed tomography. Skeletal Radiol 2002; 31: 1–8. doi: 10.1007/s00256-001-0439-y 11807585

[18] Shikhare S, Singh D, Shimpi T, Peh WCG Tuberculous osteomyelitis and spondylodiscitis. Semin Musculoskelet Radiol 2011; 15: 446–58. doi: 10.1055/s-0031-1293491 22081280

[19] TL W, Huang CH, Hwang DY, et al Primary pyogenic abscess of the psoas muscle. Int Orthop 1998; 22: 41–3. 9549580 10.1007/s002640050205 PMC3619652

[20] Huff S, Gillette M, Stirton J, Peters N, Ebraheim N, Marshall MD, Peters MD;, Nicholas MD Iliopsoas abscess presenting with sacral fracture and gluteal abscess. JAAOS: Global Research and Reviews 2017; 1(Issue 9): e078 Volume. doi: 10.5435/JAAOSGlobal-D-17-00078 30211374 PMC6132312

